# Clinical characteristics and epidermal barrier function of papulopustular rosacea: A comparison study with acne vulgaris

**DOI:** 10.12669/pjms.326.11236

**Published:** 2016

**Authors:** Maosong Zhou, Hongfu Xie, Lin Cheng, Ji Li

**Affiliations:** 1 Maosong Zhou, Department of Dermatology, Xiangya Hospital, Central South University, Changsha 410008, China; 2 Hongfu Xie, Department of Dermatology, Xiangya Hospital, Central South University, Changsha 410008, China; 3 Lin Cheng, State Key Laboratory of Ophthalmology, Zhongshan Ophthalmic Center, Sun Yat-sen University, 54 South Xianlie Road, Guangzhou, 510060, Guangdong, P. R. China; 4 Ji Li, Department of Dermatology, Xiangya Hospital, Central South University, Changsha 410008, China

**Keywords:** Papulopustular rosacea, Acne vulgaris, Epidermal barrier function, Transepidermal water loss

## Abstract

**Objective:**

To evaluate the clinical characteristics and epidermal barrier function of papulopustular rosacea by comparing with acne vulgaris.

**Methods:**

Four hundred and sixty-three papulopustular rosacea patients and four hundred and twelve acne vulgaris patients were selected for the study in Xiangya Hospital of Central South University from March 2015 to May 2016. They were analyzed for major facial lesions, self-conscious symptoms and epidermal barrier function.

**Results:**

Erythema, burning, dryness and itching presented in papulopustular rosacea patients were significantly higher than that in acne vulgaris patients (*P*<0.001). The clinical scores of erythema, burning, dryness and itching in papulopustular rosacea patients were significantly higher than those in acne vulgaris patients (*P*<0.001). The water content of the stratum cornuem and skin surface lipid level were both significantly lower in papulopustular rosacea patients than that of the acne vulgaris patients (*P*<0.001) and healthy subjects (*P*<0.001); Water content of the stratum cornuem and skin surface lipid level were higher in acne vulgaris patients in comparison with that of healthy subjects (*P*>0.05, *P*<0.001; respectively). Transepidermal water loss was significantly higher in papulopustular rosacea patients than that of acne vulgaris patients and healthy subjects (*P*<0.001); transepidermal water loss was lower in skin of acne vulgaris patients than that of healthy subjects (*P*<0.001).

**Conclusion:**

Erythema, burning, dryness and itching are the characteristics of papulopustular rosacea, which makes it different from acne vulgaris. The epidermal barrier function was damaged in papulopustular rosacea patients while not impaired in that of acne vulgaris patients.

## INTRODUCTION

Papulopustular rosacea is defined as a skin disease with prolonged flush, persistent erythema and repeating papules or pustules accompanied with drying, itching and burning sensations of the affected skin.^1^ Epidermal barrier function was impaired in papulopustular rosacea which showed reduced resistance against irritants and allergens. Rosacea is among the highest mobility in Indo-Eurasians.^2^ And it is increased from South Europe to North Europe with incidence 2.2%, 10% and 22% in Germany, Sweden and Estonia, respectively.^3^-^5^ Rosacea usually starts among adult females. Although the pathogenesis of rosacea is unknown, there are some advances to illustrate the disease. Recent advances point to the importance of skin-environmental interactions which involves not only physical and chemical factors, but also microbial factors.^6^ The impaired skin barrier function and the activated innate immunological defense are the major connected pathology contributing to the persistent inflammatory response in the affected skin.^7^ The disease is also modulated by the endogenous elements such as neurovascular factors, drugs, and psychological factors. A lower stimulating threshold corresponds to higher transepidermal water loss (TEWL) and lower stratum corneum hydration. With the identification of atopic dermatitis, the epidermal barrier deterioration in rosacea patients remains restricted to facial skin.^8^ But papulopustular rosacea is often misdiagnosed as acne vulgaris in clinic.

Acne vulgaris is a chronic inflammatory dermatosis of the pilosebaceous follicles, characterized by comedones, papules, pustules, cysts, nodules, and scars. The etiology of acne vulgaris is not fully clarified yet. The pathogenesis of acne vulgaris is known to be multimodal, including ductal hypercor-nification, enhanced sebaceous gland activity, colonization by Propionibacterium acnes and inflammation.^9^ Acne vulgaris happens primarily in the seborrhoeic areas, such as the face, neck, chest or back. In clinic, the signs and symptoms of papulopustular rosacea and acne vulgaris look alike, making them misdiagnosed for each other. Differential diagnosis is necessary to distinguish the two diseases. Therefore, in an effort to distinguish papulopustular rosacea from acne vulgaris, we summarized and compared the primary clinical features of the two diseases, and compared their epidermal barrier function.

## METHODS

### Subjects

The study was carried out in the Xiangya Hospital, Department of Dermatology, at the Central South University, after approval from the ethics committee of Xiangya Hospital. All the participants gave informed consent. The study was carried out from March 2015 to May 2016. The study enrolled 463 papulopustular rosacea female patients, the age of them ranged from 20 to 50 years old with mean age of 29.8±6.7 years, 412 age-matched acne vulgaris female patients and 400 age-matched healthy female individuals.

### Main observation index and evaluation criteria

**Fig.1:**
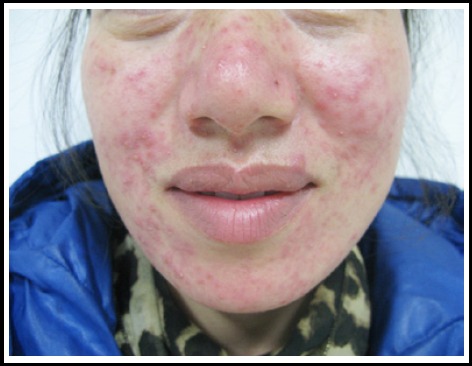
Papulopustular rosacea, tipical lesions such as erythema, papules and pustules on the face.

**Fig.2:**
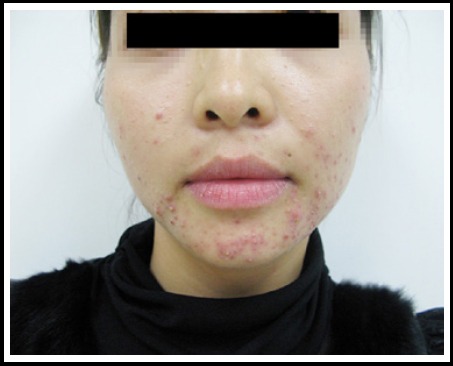
Acne vulgaris, tpical lesions such as comedo, papules and pustules on the face.

Rosacea was diagnosed by dermatologist according to the America rosacea classification standards,^1^ which is a papulopustular rosacea type: persistent erythema, transient papules or pustules, and more inflammatory rosacea subtype (Fig.1). Acne vulgaris was diagnosed according to the Pillsbury’s diagnostic criteria: (Fig.2). Exclusion criteria are as follows.

1. Retinoic acid system drugs administration within six months, or treated papulopustular rosacea or acne vulgaris (for example: antibiotics) for four weeks, or internal or external prescribed drugs treatment (retinoic acid, antibiotics) within two weeks, etc.;
2. Skin grinding dermabrasion, superficial chemical peeling surgery or laser revascularization in patients within two months;
3. Severe systemic diseases or concurrent other dermatological diseases.

The major cutaneous lesions of the two diseases were compared and scored. The assessment of lesion sites and features were done by two dermatologists at the same time. The scoring of facial self-conscious symptoms such as erythema, burning, drying, itching are defined as follows.^10^ 0=none, 1=slight, 2=mild, 3=moderate, 4=severe. The epidermal barrier function was measured on the cheek of all the subjects, including water content of the stratum cornuem, skin surface lipid level and TEWL. They were measured by Skin analysis SHP88 (Courage & Khazaka electronic GmbH, Germany). The test site was one centimeter away from the right side of the nose on the cheek. Each subject was measured three times and the values were averaged to get the mean value. Patients were acclimated for 30 min in an environmental room under standard conditions with no direct sunlight and no wind (21±1°C and 50±10% relative humidity) prior to any measurements. The subjects were instructed no cosmetics for at least 12 hours. The skin lesions were marked with a surgical marker to ensure that the measurement probes and the tapes were consistently applied to the same area.

### Statistical methods

Demographic and baseline characteristics of patients were analyzed using SPSS 19.0 software (SPSS Inc., Chicago, IL). All the numerical variables in this article were presented as mean±standard deviation (SD) (±S). Two-sided t-test was used on numerical variables. The categorical variables were compared with Pearson’s Chi-Square test. A P value < 0.05 was considered statistically significant.

## RESULTS

### Lesion Sites

The lesion sites of papulopustular rosacea and acne vulgaris are listed in Table-I. Papulopustular rosacea occurred at forehead (56.2%), cheeks (86.7%), eyelids (5.0%), nose (81.9%) and perioral (64.1%). The incidence of lesions on cheeks and nose were apparently higher than that of forehead, eyelids and perioral. For acne vulgaris, the lesions were mainly located at forehead and cheeks (74.8% and 83.2%, respectively). The incidence of lesions on nose and perioral was significantly higher in papulopustular rosacea compared with acne vulgaris (χ^2^=115.03, *P*<0.001; χ^2^=182.76, *P*<0.001). However, the incidence of lesion at forehead was significantly higher in acne vulgaris than papulopustular rosacea (χ^2^ =33.08, *P*<0.001).

**Table-I:** The lesion sites comparison between papulopustular rosacea and acne vulgaris.

Lesion Sites	Papulopustular Rosacea (463 females)	Acne vulgaris (412 females)
Forehead	260(56.2)	308(74.8)
Cheek	401(86.7)	343(83.3)
Eyelid	23(5.0)	17(4.1)
Nose	379(81.9)	195(47.3)
Perioral	297(64.1)	138(33.5)
Total	463	412

### Clinical manifestations

The clinical manifestations of papulopustular rosacea patients and acne vulgaris patients are shown in Table-II and Table-III. Erythema (persistent redness) happened at 85.0% papulopustular rosacea patients, which was apparently higher than that in acne vulgaris patients (23.8%). Comedone was not observed in papulopustular rosacea patients but occurred in 51.7% acne vulgaris patients. Inflammatory papule and pustule were common characteristics in two diseases. The subjective symptoms, such as burning, dryness and itching sensation occurred at 74.5%, 69.5% and 67.4% of papulopustular rosacea patients, which were significantly higher than acne vulgaris patients (23.1%, 27.2% and 21.6%). The clinical scores of erythema, burning, dryness and itching in papulopustular rosacea patients were 2.53±1.39, 1.33±0.98, 1.09±0.95 and 0.93±0.82, respectively, which were significantly higher than those happened in acne vulgaris patients (t=27.87, *P*<0.001; t=18.69,*P*<0.01; t=13.29, *P*<0.001; t=13.29, *P*<0.001). The data were shown in Table-III.

**Table-II:** Clinical manifestations comparison between papulopustular rosacea and acne vulgaris.

Clinical Manifestations	Papulopustular Rosacea (463 females) n (%)	Acne Vulgaris (412 females) n (%)
Erythema	395 (85.0)	98(23.8) *
Comedo	0(0.0)	213(51.7) *
Papules	463(100.0)	412(100.0)
Pustules	432(93.3)	392(95.1)
Burning	345(74.5)	95(23.1)*
Drying	322(69.5)	112(27.2)*
Itching	312(67.4)	89(21.6)*

* ***Note:*** represents P<0.05.

**Table-III:** Clinical score comparison between papulopustular rosacea and acne vulgaris.

Clinical Scores	Papulopustular Rosacea(463)	Acne Vulgaris (412)
Erythema	2.53±1.39	0.41±0.82*
Burning	1.33±0.98	0.30±0.62*
Drying	1.09±0.95	0.36±0.66*
Itching	0.93±0.82	0.26±0.54*

* ***Note:*** represents P<0.05. papulopustular rosacea compared with acne vulgaris

### Epidermal barrier function

Our study demonstrated that water content of the stratum cornuem and skin surface lipid level were significantly lower in papulopustular rosacea patients test sites in comparison with acne vulgaris patients (t=17.32, *P*<0.001; t=15.69, *P*<0.001; respectively) and healthy patients (t=6.66, *P*<0.001; t=10.21, *P*<0.001; respectively). Water content of the stratum cornuem and skin surface lipid level were higher in acne vulgaris in comparison with healthy subjects (t=1.16, *P*>0.05; t=5.49, *P*<0.001; respectively). TEWL was significantly higher in papulopustular rosacea sites in comparison with that of acne vulgaris (t=14.08, *P*<0.001) and healthy subjects (t=8.26, *P*<0.001); TEWL was lower in acne vulgaris compared with healthy subjects (t=-5.19, *P*<0.001) (Table-IV).

**Table-IV:** Epidermal barrier function comparison between papulopustular rosacea, acne vulgaris and healthy.

	Papulopustular Rosacea (n=463)	Acne vulgaris (n=412)	Healthy (n=400)
Water content of stratum cornuem (A U)	45.65±8.55	55.07±7.40	52.3±6.18
Skin surface lipid level (µg/cm^2^)	41.13±21.69	66.69±27.32	56.67±18.63
TEWL (g/hm^2^)	12.08± 4.64	8.30±3.04	9.54±3.28

## DISCUSSION

Our study aimed to investigate the primary clinical manifestations of papulopustular rosacea and acne vulgaris, and found similarities and differences between the two diseases. Papulopustular rosacea and acne vulgaris had a lot of things in common. For instance, both of them are dermatoses that affect the face, have papule and pustule lesions in face, and may have genetic traits. However, the pathogenesis of the two diseases is notably different. Papulopustular rosacea is a chronic facial dermatosis which presumed neurovascular dysregulation and neurogenic inflammation as pathogenic key factors.^11^ And it manifests almost exclusively as facial inflammatory dermatosis and is characterized by erythema, telangiectasia, papule and pustule. However, acne vulgaris is usually caused by follicular hyperkeratinization and sebaceous hypersecretion, and is often deteriorated by Propionibacterium acne, immune and inflammatory responses.^9^ So the major clinic manifestations of acne vulgaris are papule and pustule.

In our study, the clinical characteristics of papulopustular rosacea and acne vulgaris were distinctly different. Significantly higher incidence of lesions on nose and perioral in papulopustular rosacea patients was observed comparing with acne vulgaris patients. Erythema and self-conscious symptoms (burning, drying and itching) were distinctive manifestations of papulopustular rosacea compared with acne vulgaris. Erythema is featured by symmetrical lesions on forehead, nose, cheeks and chin in papulopustular rosacea, whereas, it is a restricted lesion formed by distensible or symphysic inflammatory papules in acne vulgaris. And they were able to help us to make a differential diagnosis from acne vulgaris. Therefore, for those patients who are diagnosed as acne vulgaris, if they have erythema or self-conscious symptoms, we should consider the possibility of papulopustular rosacea or merging rosacea.

In addition, the skin is a natural shield to help the body resist the external environment invasion as well as prevent moisture loss. The skin barrier is composed of cuticle, sebum membrane, structured lipids, natural moisturizing factor, etc. The skin surface lipid level, water content of stratum cornuem, TEWL are widely used to reflect skin barrier function.^12^,^13^ Any decline of structured lipids including changes of the number or the composition proportion will directly affect the epidermal barrier function. Once the epidermal barrier function is impaired, it will lead to a drop of skin natural moisturizing factor and elevations of TEWL.^14^

Papulopustular rosacea and acne vulgaris are two common chronic inflammatory facial diseases. They have similar lesions and are usually been misdiagnosed to each other in clinic. Nevertheless, the pathogenesis of the two diseases is notably different. For papulopustular rosacea, the etiology is not very clear. Some studies speculated^11^,^15^ it is due to vasomotor or neurological function disorders and capillary long-term expansion resulting from various harmful factors. Recent accumulating studies showed that the skin barrier repair plays an important role in curing papulopustular rosacea. In our study, we found decreased epidermal lipids and water content and increased TEWL in papulopustular rosacea patients in comparison with acne vulgaris and healthy subjects. Wu et al. had also found similar results with increased TEWL and decreased corneous layer water content in rosacea patients.^16^ But Raghallaigh et al.^17^ found epidermal oil content was slightly higher in papulopustular rosacea patients in comparison to healthy subjects with no significance (P>0.05). In our study, we had decreased epidermal oil content and it is probably because the healthy controls we enrolled had relatively higher oily skin ratios. We detected declined epidermal oil content in papulopustular rosacea patients and increased skin water content and oil content in acne vulgaris patients. We speculated that the skin barrier function of papulopustular rosacea was impaired, as reflected by facial drying and itching sense in papulopustular rosacea. It also suggested that moisturizers should be applied to improve dry sensation for papulopustular rosacea patients. In addition, we found that TEWL is lower and skin water content was higher in acne vulgaris compared with healthy subjects, suggesting that the skin barrier function of acne vulgaris was not impaired. The lower TEWL can be explained by retained moisture resulting from sebaceous hypersecretion.

## CONCLUSION

To sum up, our results showed that erythemas, burning, drying and itching are the clinical features of papulopustular rosacea, which differ from acne vulgaris. The epidermal barrier function was impaired in the face of papulopustular rosacea patients. In addition, we did not find impaired epidermal barrier function in acne vulgaris patients. The clinical characteristics and impaired skin barrier function will benefit us to make the right diagnosis and give better treatment to the papulopustular rosacea patients.
